# Advances in Biomimetic Scaffolds for Hard Tissue Surgery

**DOI:** 10.3390/biomimetics9050279

**Published:** 2024-05-08

**Authors:** Ryszard Uklejewski, Mariusz Winiecki

**Affiliations:** Department of Constructional Materials and Biomaterials, Faculty of Materials Engineering, Kazimierz Wielki University, Jan Karol Chodkiewicz Street 30, 85-064 Bydgoszcz, Poland

## 1. Introduction

Hard tissues are living mineralized tissues that possess a high degree of hardness and are found in organs such as bones and teeth (enamel, dentin, and cementum). The ultimate goal of bone and joint surgery, craniomaxillofacial surgery, oral/dental surgery or, in general, hard tissue surgery is reconstruction via the implantation of a biomaterial or a device to replace bones and/or joints affected by various diseases, traumatic damages, or deformities. The reconstruction of critical-sized loss or defects caused by trauma, tumor excision, osteoarthritis, and other bone-resorption-related diseases or disorders remains a significant challenge [1,2,3,4,5,6,7]. However, three-dimensional biomaterial scaffolds (produced by means of engineering or tissue engineering technologies) have emerged as relatively novel tools used to repair such damaged hard tissues [8,9,10,11]. Biomimetic scaffolds are designed and generated as biomaterial architectures that promote the regeneration of native tissue [12,13,14,15,16]. Hard tissue surgery scaffolds require mechanical stability in order to support the needed geometry of tissue loss or defects and facilitate external loading. Such scaffolds should provide internal microarchitecture to the tissue that is to be regenerated with an internal, interconnected porous network of effective space for the infiltration, growth, and differentiation of bone marrow mesenchymal stem cells, vasculature ingrowth, and new tissue growth, with the aim of ensuring a channel of material exchange with the external environment (delivering oxygen and other nutrients to the cells, in addition to waste removal) [17,18,19,20]. Thus, the design of such scaffolds is extremely important to the success of clinical outcomes in hard tissue surgery. The newest trend in this field is the viable bioinspired structural and functional design of tissue-mimicking 3D-printed (composite or hybrid) scaffolds with interconnected pore structures of controlled and often gradual porosity of implants, with the synergistic functions of promoting bone regeneration (often seeded with mesenchymal stromal cells and involving biomolecules and growth factors) and reducing local bacterial infections (intrinsically antimicrobial or loaded with antibiotics, peptides, antimicrobial metallic ions, and/or nanoparticles, anticancer drugs, etc.) [21,22,23,24,25,26,27,28,29,30].

This Special Issue aims to exhibit and discuss the latest advances in biomimetic scaffolds for hard tissue surgery, and it includes contributions on potential topics including, but not limited to, (1) biomimetic design strategies for scaffolds, (2) techniques for fabricating biomimetic scaffolds, (3) novel biomaterials for biomimetic scaffolds, (4) the biodegradability design of biomimetic scaffolds, (5) the surface functionalization of biomimetic scaffolds, and (6) clinical applications of biomimetic scaffolds.

This Special Issue presents eleven contributions submitted by scholars with renowned backgrounds in scaffolds design, fabrication, functionalization, or clinical applications [31,32,33,34,35,36,37,38,39,40,41,42,43,44,45,46,47,48,49,50]; four of these contributions are valuable review articles, and seven are original new research articles. Thematically, according to the area of application, the contributions can be grouped into articles concerning the following:

- Biomimetic scaffolds for bone and joint surgery;
- Biomimetic scaffolds for maxillofacial surgery and oral surgery;
- Biomimetic scaffolds for general surgery (i.e., hard- and soft tissue surgeries).

## 2. Overview of the Published Papers

Within the topic of biomimetic scaffolds for bone and joint surgery, two review articles highlight the advances that have been made in biomimetic scaffolds. The first review [Contribution 1] regards biomimetic silk-based materials and scaffolds for tissue engineering, with a focus on skeletal tissues, whereas the second review [Contribution 2] explores the background of currently used designs in resurfacing arthroplasty (RA) endoprostheses and presents a new approach for their component fixation via a biomimetic multi-spiked prototype scaffold.

Branković et al. (2024) [Contribution 1] review the latest research related to the potential applications of spider silk and silk-based biomaterials in reconstructive and regenerative medicine and tissue engineering, with a focus on musculoskeletal tissues. The structure and properties of spider silk, along with natural spider silk synthesis and the further advanced recombinant production of spider silk proteins, are reviewed. Research insights into possible spider silk structures, such as fibers (1D), coatings (2D), and 3D constructs, including porous structures, hydrogels, and organ-on-chip designs, are presented, with a review of applications of silk-based materials and scaffolds in musculoskeletal tissue engineering. These include bone and cartilage in addition to muscle and tendon, as well as the advanced design and precise engineering of artificial skin and vascular tissues and the design of bioactive silk-based biomaterials for smart medical implants and controlled drug delivery systems.

Uklejewski et al. (2024) [Contribution 2] take the over-hundred-year-long history of RA into scope and discuss how the designs of RA endoprostheses have evolved via a variety of designs of endoprosthesis components, different choices of materials used, and changes in methods of fixation in bone. The milestones of past design generations of RA endoprostheses are chronologically discussed along with critical insight into contemporary hip RA endoprostheses designs and their failure scenarios. As pointed out by the authors, coupled with technological advancements is the need for innovations directed towards more biomimetic designs that have materialized with the first biomimetic fixation for the RA endoprostheses being introduced. This new design type of completely cementless and stemless RA endoprostheses of knee joints and hip joints (and other diarthrodial joints), where endoprosthesis components are embedded in the surrounding bone via the prototype biomimetic multi-spiked connecting scaffold (MSC-Scaffold), initiates the first at-all generations of biomimetic endoprostheses of diarthrodial joints [44].

Of the research articles published in Part I, on biomimetic scaffolds for bone and joint surgery, two [Contributions 3 and 4] concern biomimetic design strategies for scaffolds, as well as techniques for fabricating biomimetic scaffolds, and three are concerned with novel biomaterials for biomimetic scaffolds. The personalized biomimetic scaffold for the restoration of long-bone segmental defects is the subject of Contribution 3, and the drug-releasing biomimetic scaffold for bone tissue repair is the subject of Contribution 4. Both articles present the development and characterization of 3D constructs with biological verification. Among the novel materials for biomimetic scaffolds, the three consecutive original research articles present new composite material mimicking the natural bone structure for bone scaffolds [Contribution 5], advances in ceramic biocomposites of good biomimetics of human bone composition and the means by which they are applicable in bone scaffolds [Contribution 6], and a novel biomimetic highly porous material as a scaffold with osteointegration potential [Contribution 7].

Popkov et al. (2023) [Contribution 3] propose a method for the restoration of long-bone segmental defects with the use of a bioactive degradable 3D-printed scaffold. The porous implant of a gyroid-like triply periodic minimal surface (TPMS) cellular structure was designed, fabricated via the fused deposition modeling (FDM) additive technology of ε-polycaprolactone (PCL), and coated with hydroxyapatite (HA). These implants were experimentally implanted in laboratory sheep to fill a 20 mm long segmental tibial defect, and radiological examinations demonstrated evident reparative bone tissue regeneration occurring from the proximal and distal bone fragments in the third week after surgery, the ingrowth of bone tissue into the cylindrical PCL-HA implant from the adjacent bone ends, and periosteal structures from day 7 of the study, in addition to the implant cellular structure filled with newly formed bone tissue on day 30 of the postoperative day. This in vivo study proved that the personalized biomimetic scaffold proposed provides stimulation of reparative osteogenesis and osseointegration in a single-implant bone block and is suitable for the regeneration of long-bone segmental defects.

Ensoylu et al. (2023) [Contribution 4] present the results of producing a borate-based 13-93B3 bioactive glass composite scaffold mimicking native bone tissue. In their work, hexagonal boron nitride hBN nanoparticles were included directly inside the bioactive glass matrix, and dense three-dimensional scaffolds were fabricated using the polymer foam replication method. The structural, mechanical, and biological performance of the scaffolds was investigated, and the drug delivery properties of the scaffolds loaded with gentamicin and fluorouracil were explored. The results indicate that the hBN nanoparticles, up to a certain concentration in the glass matrix, improved the mechanical strength of the glass scaffolds, while their presence enhanced the in vitro hydroxyapatite-forming ability of bioactive glass composites and accelerated the drug release rates of the system. The authors conclude that bioactive glass/hBN composite scaffolds that mimic native bone tissue could be used for bone tissue repair and regeneration applications.

Matos et al. (2023) [Contribution 5] investigated the potential of composites produced from polyvinylpyrrolidone (PVP) nanofibers containing mesoporous bioactive glass (MBG) 80S15 nanoparticles by the electrospinning technique to be used in bone tissue engineering. These polymeric scaffolds revealed the absence of cytotoxic effects on Saos-2 cells and enhanced bioactivity considering the rapid formation of hydroxycarbonate apatite (HCA) when exposed to simulated body fluid (SBF). Their degradation and swelling assays showed an ability to tailor their properties by varying the amount of MBG powder incorporated or the cross-linking properties applied, which also translates into the bioactivity (bone-bonding potential) of the composites. The authors conclude that, considering their inherent properties, the composites produced can be used for bone scaffolds because they not only reveal a high level of biocompatibility but also a swelling capacity suitable for further development, including drug delivery.

Ferro et al. (2023) [Contribution 6] present their advancements in the development of metal ion-doped ceramic biocomposites with a high level of similarity in composition to human bone and a bone-like morphology. In the study, tricalcium phosphate-based biocomposites were designed and sintered, both dense and poly(methyl methacrylate (PMMA) induced porous, doped with combinations of metal ions of magnesium (Mg^2+^), manganese (Mn^2+^), zinc (Zn^2+^), and iron (Fe^3+^); and simultaneously reinforced (for strengthening) with tetragonal zirconia (t-ZrO_2_) and cubic zirconia (c-ZrO_2_). A detailed evaluation of their physical, mechanical, and microstructural properties was performed with an evaluation of cytocompatibility in a human osteoblast (hOB) culture. The presented results led the authors to conclude that the addition of tetragonal and cubic zirconia resulted in a significant improvement in strength—up to 22% and 55%, respectively—and the addition of PMMA-generated porosity resulted in an improvement of strength up to 30% and an improvement in interconnectivity, with excellent hOB cellular viability achieved for all biocomposites produced.

Tavarro et al. (2024) [Contribution 7] propose an innovative bioactive aerogel-based composite with piezoelectric properties to assist bone regeneration. Aerogels of hydroxyapatite (HA) nanowires with barium titanate (BT, BaTiO_3_) particles were synthesized and characterized for their physical and chemical properties, bioactivity, and in vitro cytotoxicity. The results demonstrated that the HA/BT aerogel, characterized by good bioactivity and biocompatibility, constitutes a new biomaterial with osteointegration potential, which combines the advantages of a highly porous structure such as a cell scaffold (providing osteoconductivity) and can accelerate osteogenesis and osteoinduction by the presence of surface charges induced by piezoelectric BTs; thus, it could be suitable for non-load-bearing applications, such as cavity filling.

The group of papers published in Part II, titled ‘Biomimetic Scaffolds for Maxillofacial Surgery and Oral Surgery’, includes one review article [Contribution 8] discussing the recent advances in biological scaffolds for bone formation as a new tissue engineering technique for maxillofacial surgery and one new research article [Contribution 9] on the development of a 3D biomimetic scaffold to serve as a biomechanical model of fibrous periodontal ligament behavior.

Ramezanzade et al. (2023) [Contribution 8] provide a systematic review of the current literature on the angle of reconstruction of critical-sized maxillofacial defects resulting from trauma or a benign pathologic disease, using composite allogeneic tissue engineering. This technique uses an allogenic graft as a biologic scaffold in conjunction with harvested mesenchymal stem cells and recombinant human bone morphogenic protein-2 (rhBMP-2) to create, as a custom-made graft, a favorable microenvironment for new bone formation. The discussion of its reliability and efficacy allows one to see the potential of using large-scale transplantable, vascularized, and customizable bone to reconstruct large maxillofacial bony defects as a promising alternative to current therapeutic clinical options that include extensive autogenous bone harvesting and many patient morbidities.

In their work, Gauthier et al. (2023) [Contribution 9] focus on the development of a 3D fibrous scaffold with biomechanical properties representative of those of the periodontal ligament (PDL), the fibers of which would be capable of transmitting mechanical loading to ligament cells, as it is carried out in vivo by collagen bundles. Three-dimensional fibrous polycaprolactone (PCL) scaffolds were synthesized by electrospinning and seeded with human periodontal ligament cells (PDLCs), and the behavior of the cells was observed in terms of their cellular organization and signaling under static and sinusoidal axial compressive loads. The results highlight the finding that electrospun fibrous PCL 3D scaffolds mimic the in vivo mechanical deformation of PDL collagen bundles and the transmission of load to PDLCs; therefore, they might represent an interesting (suitable) experimental model for investigating PDL mechanobiology and analyzing PDLC mechanobiological behavior.

Under the topic of scaffolds for general surgery (in Part III), one review paper [Contribution 10] discusses the challenges of mechanical property adaptation in tissue-engineered scaffolds for clinical applications, and one research article [Contribution 11] presents a novel biomimetic biomaterial for tissue engineering.

Based on a study of all the relevant papers published between the years 2021 and 2023, Johnston and Callanan (2023) [Contribution 10] provide a review of the current techniques by which the mechanical properties and biological compatibility of tissue-engineered grafts (or bioscaffolds)—promoting the repair of damaged soft and hard tissues—are enhanced via hybrid material usage, multi-layer scaffold designs, and surface-modified-type scaffolds, as well as clinically translated designs, whose uses and outcomes were published within the above-given years. From their studies, the authors draw a notable observation—that accounting for the complex range of mechanical properties present in biological tissues is generally beyond the scope of a single-material-based design, and a multifaceted approach consisting of multiple material types, layers, or surface treatment methods working in tandem is likely to yield the most successful designs. They underline the fact that, in terms of translation to clinical use, the time-sensitive nature of surgical treatment suggests that 3D printing, as a fabrication method, is a strong contender for the successful realization of these designs.

Dey et al. (2023) [Contribution 11] present results on the design and development of new gelatin-based electroconductive hydrogel scaffolds and examine the angle of their potential applications for tissue engineering scaffolds. Gelatin/poly(ethylene glycol)diglycidyl ether/chitosan (G/PEG/CH) nanocomposite hydrogels with the incorporated nanosized carbon black (CB) were prepared via a mild processing condition that included aqueous media, various polymer assembly, and cross-linking chemistry, facilitating gelation with CB nanomaterial. The synthesized nanocomposite hydrogels and underwent structural, mechanical, and thermal property characteristics. As a result, the authors concluded that these nanocomposite hydrogels, which are still in the development and optimization stages, are compositionally, morphologically, mechanically, and electrically similar to native extracellular matrixes of many tissues and show great promise for use as conducting substrates for the growth of electroresponsive cells in tissue engineering.

## 3. Conclusions

These contributions published in this Special Issue on Biomimetic Scaffolds for Hard Tissue Surgery (i.e., four valuable review articles and seven new research articles) comprehensively summarize the latest achievements in the field, with a discussion of prospects and challenges for further research. Along with different proposed and developed novel biomaterials and 3D constructs of specific complementary properties that, through their biomechanical, biostructural, biochemical, biomimetic, and functional properties, possess great potential for assisting local tissue regeneration, the contributions of this Special Issue demonstrate remarkable advancements in this interesting and dynamically developing field.

## Data Availability

No new data were created or analyzed in this study. Data sharing is not applicable to this article.
